# Mink, SARS-CoV-2, and the Human-Animal Interface

**DOI:** 10.3389/fmicb.2021.663815

**Published:** 2021-04-01

**Authors:** Florence Fenollar, Oleg Mediannikov, Max Maurin, Christian Devaux, Philippe Colson, Anthony Levasseur, Pierre-Edouard Fournier, Didier Raoult

**Affiliations:** ^1^IHU-Méditerranée Infection, Marseille, France; ^2^IRD, AP-HM, SSA, VITROME, Aix Marseille University, Marseille, France; ^3^IRD, AP-HM, MEPHI, Aix Marseille University, Marseille, France; ^4^CNRS, Grenoble INP, CHU Grenoble Alpes, TIMC-IMAG, Université Grenoble Alpes, Grenoble, France

**Keywords:** SARS-CoV-2, COVID-19, mink, ferret, anthropo-zoonosis, outbreak

## Abstract

Mink are small carnivores of the Mustelidae family. The American mink is the most common and was imported to Europe, Asia, and Latin America for breeding, as its fur is very popular. Denmark, the Netherlands, and China are the biggest producers of mink. Mink farms with a high population density in very small areas and a low level of genetic heterogeneity are places conducive to contagion. The mink’s receptor for SARS-CoV-2 is very similar to that of humans. Experimental models have shown the susceptibility of the ferret, another mustelid, to become infected with SARS-CoV-2 and to transmit it to other ferrets. On April 23, 2020, for the first time, an outbreak of SARS-CoV-2 in a mink farm was reported in the Netherlands. Since then, COVID-19 has reached numerous mink farms in the Netherlands, Denmark, United States, France, Greece, Italy, Spain, Sweden, Poland, Lithuania, and Canada. Not only do mink become infected from each other, but also they are capable of infecting humans, including with virus variants that have mutated in mink. Human infection with variant mink viruses with spike mutations led to the culling in Denmark of all mink in the country. Several animals can be infected with SARS-CoV-2. However, anthropo-zoonotic outbreaks have only been reported in mink farms. The rapid spread of SARS-CoV-2 in mink farms raises questions regarding their potential role at the onset of the pandemic and the impact of mutants on viral fitness, contagiousness, pathogenicity, re-infections with different mutants, immunotherapy, and vaccine efficacy.

## Introduction

Since the start of the pandemic, the involvement of animals has been mentioned in its occurrence. Although the possibility of other sources has been suggested, of the first 41 people hospitalized with pneumonia who received a confirmed and official diagnosis of SARS-CoV-2 infection on January 2, 2020, two-thirds were linked to the Huanan Seafood wholesale market, Wuhan, Hubei, China (Hui et al., 2020). In addition, 33 of 585 (5.6%) environmental samples obtained in the market, which was billed as a live animal and seafood market, indicated evidence of SARS-CoV-2 according to the Chinese Center for Disease Control and Prevention (http://www.chinacdc.cn/yw_9324/202001/t20200127_211469.html, Accessed March 03, 2021). In addition, with coronaviruses such as SARS-CoV and MERS-CoV circulating mainly among animals, a potential link between the pneumonia epidemic and the market has been suspected, as the virus may have been transmitted to humans by an animal (Liu and Saif, 2020).

Subsequently, bats and pangolins gained attention as coronaviruses closely related to SARS-CoV-2 were detected in them (Zhang et al., 2020; Zhou et al., 2020a,b). More recently, attention has focused on mink, and more specifically farmed mink. This interest peaked when mutants of SARS-CoV-2 were transmitted from farmed mink to humans, leading to the culling of many millions of mink, in fear that the latter would transmit strains more virulent, more contagious, or resistant to vaccines in development (Larsen and Paludan, 2020; Oude Munnink et al., 2021).

Our main objective is to review the present knowledge on mink, including their ability to be infected with SARS-CoV-2, to infect humans with variants, and the impact this may have.

## Ecology of Mink Worldwide

### The Mink, a Member of the Family Mustelidae

The term “mink” is usually applied to at least two different animals from the family Mustelidae; the European mink (*Mustela lutreola*) and the American mink (*Neovison vison*). The two minks, as in other mustelids, are small carnivores with characteristically elongated bodies. Mustelids contain around 60 known species, including, in addition to mink, otter, ferret, polecat, marten, ermine, badger, sable, and wolverine (Canuti et al., 2020). Several mustelids, including mink, sable, and ermine, have been hunted since prehistoric times for their fur. One of the major economic stimuli for Russian expansion into Siberia and Far East, as well as French and English expansion in North America, was the abundance of furbearers in these territories.

Some mustelids have been domesticated. The ferret (*Mustela putorius furo*) and the tayra (*Eira barbara*) are kept as pets. The ferret has been kept in Europe for a long time; the famous Leonardo da Vinci painting of the “Lady with an Ermine” holds in her hands, the albino form of domesticated ferret. As for mink, its aggressive behavior prevents their use as pets, but their fur has led to their breeding on mink farms. In recent centuries, however, fur farming, notably of American mink, has also become widespread, and provides the majority of the fur brought to market.

### Feral Mink

Mink are semi-aquatic, living along waterways. They are solitary, and rather sedentary.

The European mink was widely distributed over most of continental Europe a century ago (Karath, 2017; Skorupski, 2020). Currently, only a few thousand (an estimated 5,000 individuals) persist in the wild in Spain, France, and the Danube delta. In Russia, sightings have become so rare that the species is considered to be on the brink of extinction (Skorupski, 2020). The European mink now occupies less than 3% of its former habitat. It is currently considered one of the most endangered mammal species in the world (Skorupski, 2020). Reintroductions of populations have been carried out in Estonia, Germany, and Spain (Karath, 2017; Skorupski, 2020). Even if the disappearing habitat and hunting may play a role in this situation, the main cause is the introduction of the invasive American mink (Karath, 2017; Skorupski, 2020).

The American mink is native to North America, where it is found throughout Canada and most of the United States, except in Arizona and the arid parts of California, Nevada, Utah, New Mexico, and western Texas. First imported in Europe by fur farmers for their superior pelt in the 1920s, some animals escaped and thrived in the wild. Thus, escapees have established populations in England, France, Germany, Iceland, Ireland, Norway, Poland, Scotland, and Sweden (Bevanger and Henriksen, 1995; Lariviere, 2021). Bigger, more adaptable, more aggressive, and more fertile, they have simply replaced the native species (Karath, 2017).

The ability of the species to colonize new habitats is excellent, and it is estimated that all of Sweden was invaded in approximately 35 years (Gerell, 1967). *Neovison vison* was also brought to South America for fur farming in the 1930s, and numerous populations have been recorded in the wild since 1960–1961 (Lariviere, 2021).

### Mink Farms

**Figure 1:**
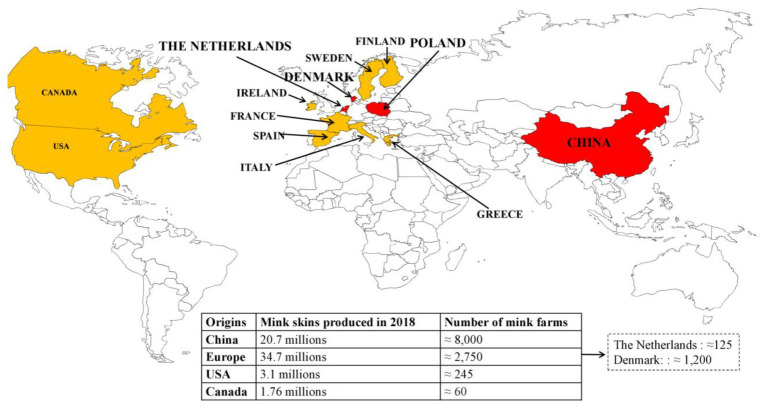
Distribution of the largest producers and the main mink farms worldwide. The four biggest producers are in red, the following ones are in orange.

The American mink is currently the most important species in fur-farming operations (Tomson, 1987). The main fur producing countries are Denmark, the Netherlands, Poland, and China, where the first mink farms were established in the 1950s (Figure 1). China is the leading market for fur. According to the Fur Commission USA, in 2012, the United States exports of mink pelts to China reached a record high of $215.5 million (https://furcommission.com/u-s-mink-manufacturers-eye-growing-chinese-demand-for-fur/p, Accessed February 03, 2021). At the same time, China doubled its domestic mink production, contributing to a record 80 million pelts produced worldwide.

Currently, the annual production of Chinese mink is over 20 million (20.7 in 2018); the economic benefits are considered significant (Gong et al., 2020). Overall, 95% of fur farms are concentrated in the Northern provinces: Shandong (greatest concentration), Liaoning, Heilongjiang, Jilin, and Henan (Gong et al., 2020). According to the European Centre for Disease Prevention and Control, Europe has ≈2,750 mink farms. There are ≈ 1,200 mink farms in Denmark (Hammer et al., 2021); ≈ 125 in the Netherlands, with an average of 5,000 female breeding animals (Oreshkova et al., 2020); ≈ 900 in Finland; and ≈ 300 in Poland, where data must be adjusted, as numerous mink farms have been closed in the past 4 years. European production was 34.7 million mink pelts in 2018. In the United States, there are ≈ 245 fur farms that produced 3.1 million pelts in 2018. In Canada, 1.76 million mink pelts were produced in 2018 on ≈ 60 farms. In Russia, according to the Russian National Association of Fur Breeders, published in the official journal of the Russian Federal Service for Veterinary and Phytosanitary Supervision, “Veterinaria i zhizn,” 22 enterprises in 14 regions of the country are engaged in breeding mink, with a broodstock of about 300 thousand individuals.

Female mink are bred in March and whelp in May (Tomson, 1987). The young are weaned in July and are then placed in individual cages to prevent fighting and damage to the fur. They have a fur molt in early fall and are killed (pelted) when their pelt is “prime”; that is, when the pigment has migrated from skin follicles into hair shafts. The young that are desired for breeding are held over for the next breeding season. Farm mink are usually fed with a commercially produced wet feed, consisting of a mix of animal by-products and slaughter offal, e.g., by-products from the fishing and meat industries and plant origin, such as corn gluten meal, soybean oil, and extruded cereals (Lyhs et al., 2019).

Hundreds to thousands of cages in close proximity are frequently housed in a small area, in a single shelter or building. Disease problems are those caused by intensive farming practices, marginal nutrition, and poor sanitation. Contagion is facilitated by the proximity of animals and their low genetic diversity (reproduction using a few males selected for their fur).

## Mink and Contagious Diseases

### Well-Known Contagious Diseases

Several microorganisms can affect mustelids (Canuti et al., 2020). The most studied are those commonly found in mink on fur farms. Aleutian disease, considered as the “most serious” infectious disease affecting farmed mink, is caused by a highly contagious and environmentally resistant amdoparvovirus. The disease is characterized by a chronic wasting syndrome involving disruption of the immune system, with an impact on mortality and reduced mink reproduction (Gong et al., 2020). Distemper is caused by a morbillivirus. It is fatal to unvaccinated mink. Dogs are the common source. However, the role of wildlife reservoirs, such as the fox, has been observed during a major epidemic in mink farms in Denmark. Most mink farms have outer perimeter fences to exclude feral canines from their mink (Trebbien et al., 2014). Mink enteritis is caused by a highly contagious virus closely related to feline panleukopenia virus and canine parvovirus type 2 (Wilson et al., 2015). In outbreaks, mortality is very high, reaching 75% in weaned mink. The most common bacterial diseases include type C botulism and hemorrhagic pneumonia caused by specific strains of *Pseudomonas aeruginosa* (Wilson et al., 2015). Mink are also often affected by coccidiosis (Tomson, 1987).

### Emergent Contagious Diseases

Since 2010, new epidemics have been reported in mink farms. In 2011, a new orthoreovirus was reported in mink on a farm in Hebei Province, China. Almost all mink were infected, with an estimated mortality of <5% (Lian et al., 2013). In 2014, an invasive outbreak of swine pseudorabies occurred in mink on a farm in Shandong Province, China, with a mortality rate of 87% (3,522/4,028; Wang et al., 2018). In 2014, Newcastle disease, due to avian paramyxovirus serotype-1, was described in mink on a farm in Heilongjiang province, China, responsible for hemorrhagic encephalitis and pneumonia, with a death rate of 95% in the 9% of affected mink (Zhao et al., 2017). In 2015, epidemics due to an H5N1 avian influenza virus were reported in two mink farms, 200 km apart, in Northeast China. The death rates were 56% (128/230) and 64% (242/376; Jiang et al., 2017).

Interestingly, many emerging infectious diseases reported in mink have high zoonotic potential. It seems that farmed mink are susceptible for the infections of different vertebrate, including birds, pork, and humans.

## Zoonotic Epidemiology of SARS-CoV-2 Infection

Early investigations showed that human SARS-CoV-2 is closely related to the MN996532_raTG13 and RmYN02 coronaviruses from the Chinese horseshoe bats *Rhinolophus affinis* and *Rhinolophus malayanus*, respectively (Zhou et al., 2020a,b). Because of the lack of evidence for direct transmission of bat coronaviruses to humans (Afelt et al., 2018) and the spillover theory of zoonotic emergence that postulates an animal reservoir at the origin of the zoonosis (Plowright et al., 2017), many research groups worldwide have attempted to identify an intermediate susceptible animal able to pass a SARS-CoV-2-like virus to humans. Snakes (*Ophiophagus hannah*) were first proposed to be the possible animal reservoir (Ji et al., 2020). After this hypothesis was refuted (Callaway and Cyranoski, 2020), the Malayan pangolin (*Manis javanica*), was in turn designated as the intermediate host (Zhang et al., 2020). However, the pangolin hypothesis was also refuted (Frutos et al., 2020b; Liu et al., 2020).

Soon after the identification of SARS-CoV-2, it was demonstrated that the viral receptor is the human angiotensin-I-converting enzyme 2 (ACE2; Qiu et al., 2020; Yan et al., 2020; Zhao et al., 2020). ACE2 is a peptidase that controls the renin-angiotensin-aldosterone system regulating blood pressure, and there is a known polymorphism of ACE2 among human populations and between other species (Cao et al., 2020; Devaux et al., 2020). To resolve the issue of the animal reservoir, investigations redoubled in intensity, based on knowledge that the ability of SARS-CoV-2 to infect an animal must depend on several factors: contact with an infected host releasing infectious particles, compatibility between the spike protein of the virus and the host receptor ACE2 of the target species, body temperature, the host species preferences of SARS-CoV-2, and the capacity of the virus to escape the immune system and restriction factors of the new host (Uzoigwe, 2020).

With the availability of published crystallographic analyses that determined which amino acids of ACE2 are essential for the viral spike protein attachment (Lan et al., 2020; Shang et al., 2020; Yan et al., 2020), one strategy for fast identification of possible SARS-CoV-2 target species was performed using *in silico* screening of species ACE2 orthologs with potential high affinity for the viral spike. This *in silico* approach predicted that a very large number of species are possibly susceptible to SARS-CoV-2, including humans (*Homo sapiens*), monkeys (*Macaca mulatta*), cats (*Felis silvestris catus*), bats (*Rhinolophus sinicus*), pangolins (*M. javanica*), turtles (*Pelodiscus sinensis*), and many others (Devaux et al., 2020; Liu et al., 2020; Luan et al., 2020; Qiu et al., 2020). A recent work scored 25 amino acids considered as important for interaction between the SARS-CoV-2 spike and ACE2, and suggested that 252 mammal and 72 bird ACE2 orthologs could serve as entry receptors for SARS-CoV-2 in animal species (Damas et al., 2020). This strongly supports the hypothesis that there is not a single intermediate host, but many susceptible species, that SARS-CoV-2 circulates between species in the ecosystem, and when the conditions for its dissemination are met (in particular, a high density of susceptible hosts), an infectious contact may be sufficient to trigger an outbreak in susceptible animal or human populations (Frutos et al., 2020a).

Gradually, *in vitro* and *in vivo* experimental infections of animals confirmed this broad spectrum of targets for SARS-CoV-2. Indeed, SARS-CoV-2 can infect a wide number of wild and domesticated species (Shi et al., 2020). Based on experimental models, monkeys (Munster et al., 2020; Rockx et al., 2020), ferrets (Schlottau et al., 2020), cats (Halfmann et al., 2020; Shi et al., 2020), as well as raccoon and dogs (Freuling et al., 2020), hamsters (Bertzbach et al., 2020), and rabbits (Mykytyn et al., 2021) are among the most susceptible species, while other species such as pigs are susceptible but poorly replicate the virus (Schlottau et al., 2020), and other species are resistant to infection, such as the mouse (Bao et al., 2020). Since then, several reports have indicated the natural infection of domestic animals, cats and dogs, by their infected owner (Leroy et al., 2020). Recently, an investigation of SARS-CoV-2 and anti-SARS-CoV-2 neutralizing antibodies in 919 pets from northern Italy was reported, which indicated that 3.3% of dogs and 5.8% of cats had measurable neutralizing antibody titers (Patterson et al., 2020). A pet ferret with digestive signs and in contact with a COVID-19 person was also detected to be positive for SARS CoV-2 in Slovenia (https://www.oie.int/wahis_2/public/wahid.php/Reviewreport/Review?page_refer=MapFullEventReport&reportid=37289, Accessed February 03, 2021).

Sporadic cases of SARS-CoV-2 infections have been reported among other members of the Felidae family, specifically tigers, lions, pumas (*Puma concolor*), and snow leopards (*Panthera uncia*) caged in zoos (Hosie et al., 2021; https://promedmail.org/promed-post/?id=8002466, Accessed February 03, 2021, https://www.aphis.usda.gov/aphis/newsroom/stakeholder-info/sa_by_date/sa-2020/sa-12/ky-snow-leopard-covid, Accessed February 03, 2021). The latter were most likely infected by zoo employee infected by SARS-CoV-2 (Oude Munnink et al., 2021).

**Figure 2:**
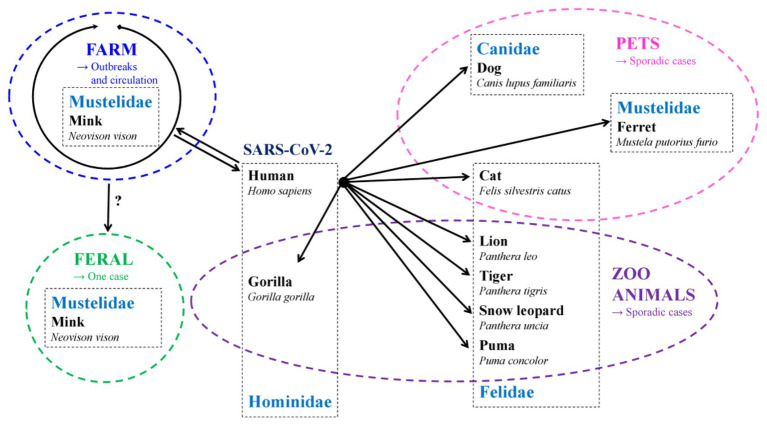
Species naturally infected with SARS-CoV-2 and the origin of transmission (→).

More recently, another member of the Hominidae family, the gorilla, was also reported to be infected with SARS-CoV-2 in a zoo. Besides ferrets, other members of the Mustelidae family such as the American mink (*Neovison vison*), are *in vivo* susceptible to SARS-CoV-2 and can transmit the virus both to other susceptible animals and to humans. Mink can be infected by SARS-CoV-2 from infected animals and humans. They can also transmit the virus to other mink and to caregivers in mink farms. The species that have been reported to be naturally infected with SARS-CoV-2 are summarized in Figure 2.

## Evidence of SARS-CoV-2 Infection in Mink and Other Mustelids

### Ferret Experimental Model

The ferret, *M. putorius furo*, is another member of the genus *Mustela* in the family Mustelidae, and can be considered as the “cousin” of the mink. It is also the domesticated form of the European polecat. Ferrets are usually considered a good animal model for viral respiratory diseases (Belser et al., 2016; Hewitt et al., 2020). Based on several experimental models, ferrets were shown to be highly susceptible to SARS-CoV-2 infection (Blanco-Melo et al., 2020; Hewitt et al., 2020; Kim et al., 2020; Shi et al., 2020). In most models, ferrets are infected with SARS-CoV-2 *via* the intranasal route to better mimic the natural route of infection in COVID-19 patients (Blanco-Melo et al., 2020; Hewitt et al., 2020; Kim et al., 2020; Shi et al., 2020). The inoculum dose varies from 5 × 10^5^ to 5.5 × 10^6^ plaque-forming units of the virus. Various SARS-CoV-2 strains have been used, including human strains [e.g., NMC-nCoV02 (Kim et al., 2020), CTan-H (Shi et al., 2020) strains, and Victoria/1/2020 SARS-CoV-2 (Ryan et al., 2021)] and environmental strains [e.g., the F13-E strain collected from an environmental sample in the Huanan Seafood Market in Wuhan (Shi et al., 2020)].

Infected ferrets usually develop mild clinical symptoms, including fever 2–8 days post-infection, reduced activity, and occasional cough (Kim et al., 2020). Clinical signs usually disappear spontaneously within 2 weeks of infection; no fatalities have been reported (Kim et al., 2020; Ryan et al., 2021). Viral RNA can be detected in nasal washes from 2 up to 20 days post-infection (Kim et al., 2020; Ryan et al., 2021), with the highest viral load occurring at 4 days [e.g., 3.8 log_10_ RNA copies/ml (Kim et al., 2020)], but also in blood, saliva, urine, and feces. Less frequently, viral RNA was detected in lungs, kidney, intestine, and fecal samples between 4 and 8 days post-infection (Kim et al., 2020). In contrast, the virus was not detected in the heart, liver, spleen, pancreas, and brain samples from these animals (Shi et al., 2020). About 2–3 weeks following infection, viral RNA was no longer detectable in nasal washes or any organs (Shi et al., 2020; Ryan et al., 2021).

Infectious viruses could be isolated in cell cultures from nasal washes, saliva, trachea, and lungs collected from 2 to 4–6 days post-infection (Kim et al., 2020). Specific immunohistopathological findings have also been reported and are summarized in Table 1 (Kim et al., 2020; Shi et al., 2020; Ryan et al., 2021). Antibodies against SARS-CoV-2 were detected in infected ferrets by ELISA and neutralization assays at 2–3 weeks post-infection (Kim et al., 2020). Serological titers ranged from 32 to 128 at that time (Kim et al., 2020). Finally, infected ferrets can also transmit SARS-CoV-2 to other ferrets, with significant animal-to-animal transmission through direct contact and the aerosol route (Table 2; Kim et al., 2020; Richard et al., 2020; Schlottau et al., 2020).

**Table 1:** Histopathological abnormalities observed in an experimental ferret model.

References	Date	Main specific immunohistopathological findings
Kim et al. (2020)	4 days post-infection	- Increased immune infiltration and cell debris in the alveolar wall, bronchial epithelium, and bronchial lumen, suggestive of acute bronchiolitis
	12 days post-infection	- Disappearance of pathological abnormalities
Ryan et al. (2021)	3–14 days post-infection	- Mild multifocal bronchopneumonia - Mild necrosis of the bronchiolar epithelial cells together with inflammatory cell infiltration of neutrophils and mononuclear cells within the bronchiolar lumina
Shi et al. (2020)	13 days post-infection	- Severe lymphoplasmacytic perivasculitis and vasculitis in lungs - Increased numbers of type II pneumocytes, macrophages and neutrophils in the alveolar septa and alveolar lumen - Mild peribronchitis

**Table 2:** Evaluation of SARS-CoV-2 transmission between ferrets.

References	Infected ferrets	Transmission method	Challenged naïve ferrets
Kim et al. (2020)	- All ferrets inoculated with SARS-CoV-2 developed fever at 2–8 days post-infection	Direct contact with other infected ferrets	- All became febrile with reduced activity 4–6 days post-contact - Most collected specimens were positive for viral RNA - Isolation of viruses from nasal washes
		Indirectly *via* aerosols → Different cages with a permeable partition	- None of the ferrets developed fever - Detection of viral RNA in nasal washes from only 2 of 6 ferrets with indirect contact - Positive serological titer (16) in only one
Schlottau et al. (2020)	- Infection of 12 ferrets intranasally with 10^5^ TCID_50_ of viral load - None developed fever or body weight loss. - Detection of virus in nasal washes in most animals by qPCR between 2 and 8 days post-infection and culture at 2–4 days post-infection - All developed neutralizing antibodies	Direct contact with other infected ferrets	- All three naive ferrets acquired SARS-CoV-2 with viral RNA detection in nasal washes over 8–21 days post-infection - Detection of neutralizing antibodies in only one contact ferret
Richard et al. (2020)	- Infection was confirmed by viral RNA shedding in nasal washes from 3 to 19 days post-infection	Direct contact with other infected ferrets	- Transmission to four of four naïve animals after 1–3 days. - Detection of viral RNA in newly infected ferrets up to 13–15 days post-infection
		Indirect transmission → Close but different cages	- Transmission to three of four naïve animals - Viral RNA was detected from 3 to 7 days post-exposure and over 13–19 days - Lower neutralizing antibody titers

### First Alert in Mink

As early as April 23 and 25, 2020, two closely situated (17 km apart) mink farms in the North Brabant province of the Netherlands, housing 21,200 animals, reported increased mortality in mid-April 2020 (Oreshkova et al., 2020; Oude Munnink et al., 2021). Animal necropsy enabled the detection of SARS-CoV-2 (Molenaar et al., 2020). Post-mortem findings also showed acute interstitial pneumonia in almost all mink examined (Molenaar et al., 2020). Overall, the impact of SARS-CoV-2 infection in mink ranged from asymptomatic to death, with a spectrum similar to humans (Molenaar et al., 2020). Despite strict quarantine measures, subsequent official investigations identified additional infected farms in the same region. In farms, animal-to-animal transmission was facilitated by high animal density. From June 6, mink from infected farms were culled. In early November, Dutch health authorities decided to cull all mink in the country and ban mink farming. In the Netherlands, the minks at non-infected farms were not culled. All animals, including those of the breeding stock, at non-infected farms were pelted, as per normal procedure in November and December. Thus, only animals at the infected farms were culled as of June, as soon as infection was detected. Mink farming in the Netherlands is banned as of January 2021.

### Epidemiology of Mink SARS-CoV-2 Infection

After the first Dutch cases in April, Denmark, the largest European mink pelt producer, reported mink farm infections in May 2020 in the Jutland region (Hammer et al., 2021). In mid-June, the Danish government imposed the culling of infected animals in infected farms. On November 4, 2020, due to the emergence of 12 cases of human infections caused by a mink SARS-CoV-2 variant (referred to as “Cluster 5”; Larsen and Paludan, 2020), a culling of all mink was decreed. In the Netherlands and Denmark, COVID-19 cases were diagnosed among farm workers before infections in mink were detected, suggesting that the animals were infected by humans. Human and mink viral strain genome sequences, although slightly different by a few mutations, clustered together (Oude Munnink et al., 2021).

In the United States of America, the outbreaks seem to be well documented and reported to the World Organization for Animal Health. The first cases of infection in farmed mink were reported in Utah on August 17, 2020, in Wisconsin and Michigan on October 8 and 9, 2020 and in Oregon on November 27, 2020, with mortality rates of mink infected by SARS-CoV-2 varying according to the farms (Table 3). To date, in addition to the Netherlands (69 mink farms), Denmark (290 mink farms), and United States (17 mink farms), SARS-CoV-2-infected farms have been reported in France (1 mink farm), Greece (22), Italy (1), Spain (1), Sweden (13), Poland (1), Lithuania (2), and Canada (2; https://www.oie.int/en/, Accessed February 03, 2021). Overall, cases of infections in mink farms have been reported in Europe and North America. No cases of COVID-19 infection in breeding mink have been diagnosed in Russia (https://www.vetandlife.ru/vizh/sobytiya/kak-v-rossii-zashchishchayut-norkovye-fermy-ot-covid-19/?sphrase_id=5987, Accessed February 03, 2021). To the best of our knowledge, no cases have been reported in China.

**Table 3:** Mortality rates of mink infected by SARS-CoV-2 according to the farms in the United States of America.

Date of the outbreak	U.S. states	Number of dead mink/Number of mink in the farm	Sources (Accessed February 03, 2021)
07/26/20	Utah	3,524/20,000 (16.3%)	https://www.oie.int/wahis_2/public/wahid.php/Reviewreport/Review?page_refer=MapFullEventReport&reportid=35412; https://promedmail.org/promed-post/?id=7692815
08/02/20	Utah	1,451/8,983 (16.2%)	https://www.oie.int/wahis_2/public/wahid.php/Reviewreport/Review?page_refer=MapFullEventReport&reportid=35412; https://promedmail.org/promed-post/?id=7692815
08/03/20	Utah	1,554/6,326 (24.6%)	https://www.oie.int/wahis_2/public/wahid.php/Reviewreport/Review?reportid=35525
08/05/20	Utah	1,119/3,643 (30.7%)	https://www.oie.int/wahis_2/public/wahid.php/Reviewreport/Review?reportid=35525
08/15/20	Utah	205/1,705 (12%)	https://www.oie.int/wahis_2/public/wahid.php/Reviewreport/Review?reportid=35525
09/07/20	Utah	146/600 (24.3%)	https://www.oie.int/wahis_2/public/wahid.php/Reviewreport/Review?page_refer=MapFullEventReport&reportid=35946
09/20/20	Utah	247/14,000 (1.8%)	https://www.oie.int/wahis_2/public/wahid.php/Reviewreport/Review?page_refer=MapFullEventReport&reportid=35946
09/24/20	Utah	59/1,500 (3.9%)	https://www.oie.int/wahis_2/public/wahid.php/Reviewreport/Review?page_refer=MapFullEventReport&reportid=35857
09/27/20	Michigan	2,000/17,000 (11.8%)	https://www.oie.int/wahis_2/public/wahid.php/Reviewreport/Review?page_refer=MapFullEventReport&reportid=35973
09/29/20	Utah	126/300 (42%)	https://www.oie.int/wahis_2/public/wahid.php/Reviewreport/Review?page_refer=MapFullEventReport&reportid=36151
09/30/20	Wisconsin	1,800/14,600 (12.3%)	https://www.oie.int/wahis_2/public/wahid.php/Reviewreport/Review?page_refer=MapFullEventReport&reportid=35973
10/08/20	Utah	373/3,000 (12.4%)	https://www.oie.int/wahis_2/public/wahid.php/Reviewreport/Review?page_refer=MapFullEventReport&reportid=36580
10/19/20	Wisconsin	2,200/22,500 (9.8%)	https://www.oie.int/wahis_2/public/wahid.php/Reviewreport/Review?page_refer=MapFullEventReport&reportid=36580
10/22/20	Utah	585/13,200 (4.4%)	https://www.oie.int/wahis_2/public/wahid.php/Reviewreport/Review?page_refer=MapFullEventReport&reportid=36580
10/22/20	Oregon	No excess mortality/12,000	https://www.oie.int/wahis_2/public/wahid.php/Reviewreport/Review?reportid=36731
10/25/20	Utah	739/38,000 (2%)	https://www.oie.int/wahis_2/public/wahid.php/Reviewreport/Review?page_refer=MapFullEventReport&reportid=36580
11/04/20	Wisconsin	3,400/No data available	https://promedmail.org/promed-post/?id=7923387
11/05/20	Wisconsin	2,000/No data available	https://promedmail.org/promed-post/?id=7923387

In Spain and Italy, infected mink farms have been suspected to have played a role in the regional spread of SARS-CoV-2. At the end of June, an outbreak of COVID-19 caused by a variant named 20A-EU1 began in the Aragon region of Spain and then rapidly spread to other European countries because of tourist travel (Hammer et al., 2021). The region where the outbreak started is known to host several mink farms where animal infections were detected. In Italy, the role of mink is also suspected, as the emergence of the D614G mutation occurred in the Lombardy region, where Italian mink farms are located.

Since August 2020, Utah has been battling outbreaks of COVID-19 in mink farms (https://www.kuer.org/health-science-environment/2020-12-15/novel-coronavirus-detected-in-a-wild-mink-near-infected-utah-fur-farm, Accessed February 03, 2021). A state veterinarian said in November that nearly 11,000 mink have died from the disease. In mid-December, a wild mink, found while federal officials were surveying the area around these farms for the virus, tested positive for the SARS-CoV-2. It is believed to be the first confirmed case in a free-ranging native animal. A report on December 13, 2020 from the United States Department of Agriculture suggests the animal acquired its infection from farmed mink. The wild animal harbored a virus that appears identical to what was seen in nearby farmed mink (https://promedmail.org/promed-post/?id=8015608, Accessed February 03, 2021).

## Mink SARS-CoV-2 Virus Phylogeny

**Figure 3:**
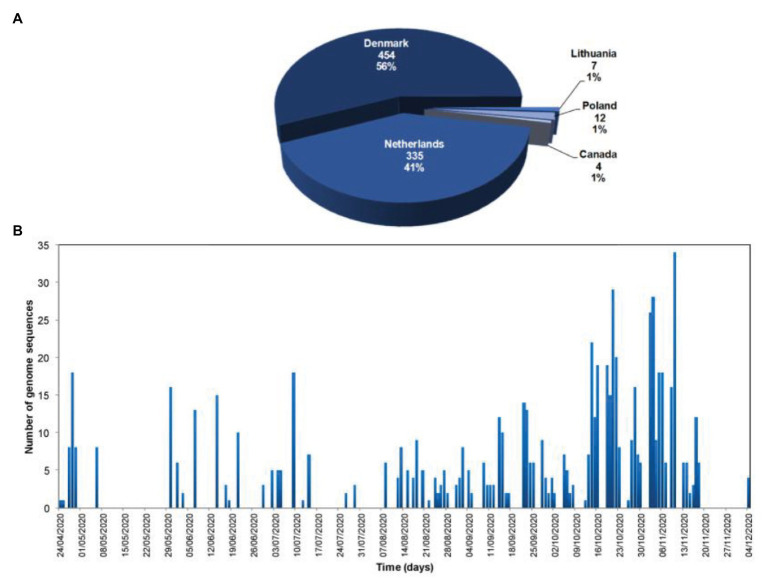
Number and geographic origin of mink SARS-CoV-2 genomes available in the Global Initiative on Sharing Avian Influenza Data (GISAID database; https://www.gisaid.org/; Accessed February 02, 2021). **(A)** Pie chart of the number of SARS-CoV-2 genome sequences from minks per country, and proportion of the total number of SARS-CoV-2 genome sequences from minks; **(B)** Temporal distribution of the number of SARS-CoV-2 genome sequences from minks per day of sample collection.

**Figure 4:**
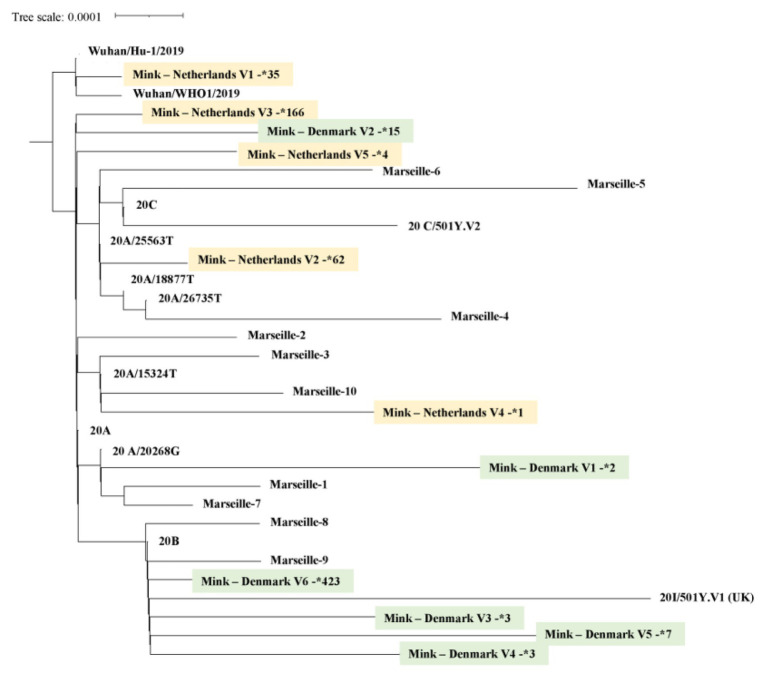
Mink SARS-CoV-2 virus phylogeny. A total of 744 SARS-CoV-2, selected from GISAID (https://www.gisaid.org/), were integrated in a phylogenetic analysis. All genomes were aligned by using MAFFT version 7 (Katoh and Standley, 2013). A phylogenetic tree was reconstructed by using IQ-TREE with the GTR model with ultra-fast bootstrap of 1,000 repetitions (Minh et al., 2020). Sequences of mink from the Netherlands are highlighted in yellow, those from Denmark in green. The number next to the star is the number of genomes available for each mink SARS-CoV-2 genotype.

Currently, 812 mink SARS-CoV-2 genomes are available in the Global Initiative on Sharing Avian Influenza Data (GISAID) database (https://www.gisaid.org/, Accessed February 03, 2021; Figure 3). Overall, there is great genetic diversity of mink viruses. Phylogenetic reconstruction (Katoh and Standley, 2013; Minh et al., 2020) based on SARS-CoV2 isolated from mink and humans has revealed distinct clades (see Figure 4). Isolates from mink were divided into five and six main groups of samples from the Netherlands and Denmark, respectively. Interestingly, we observed a common node between genomes from mink, from variants 20A.EU2-Marseille-4, Marseille-5, and Marseille-6, and from variant 20H/501Y.V2 from England. This node pointed to a common mutation in G25563U/Q57H in ORF3a of the SARS-CoV2 genome. ORF3a encodes a protein with three transmembrane domains and a large cytoplasmic domain and may play a structural role in the viral life cycle of SARS-CoV. Previous studies have also reported that ORF3a can induce apoptosis in cells and therefore may be involved in pro-apoptotic activity (Law et al., 2005; Freundt et al., 2010). Genetic diversification is linked to the adaptation of the virus to a new host. Indeed, under selective pressure from the mink immune system, new mutations can be fixed in the SARS-CoV-2 genome, and the mink SARS-CoV-2 variant virus can be introduced back into human populations.

About 170 mutations have been identified by whole genome sequencing of mink SARS-CoV-2 samples from 40 mink farms, and mink-specific mutations of SARS-CoV-2 (including a Y453F mutation in the viral spike) have been found in humans (Mallapaty, 2020). In addition, 23 mutations independently appeared at least twice in SARS-CoV-2 circulating in mink (https://www.biorxiv.org/content/10.1101/2020.11.16.384743v1, Accessed February 03, 2021). The virus appears to evolve at a faster rate in mink than in humans (a mutation every 2 weeks; Oude Munnink et al., 2021), possibly due to a phenomenon of host adaptation. Seven nonsynonymous mutations in viral genomes from mink that independently arose at least three times are plausible candidates for adaptation to transmission in mink. Among these, three mutations in the receptor binding domain of the spike protein are nonsynonymous, which raises questions about the efficacy of current vaccines in case of human infection with such strains.

## Human Cases

Human cases of infection from mink infected with SARS-CoV-2 that have mutated in mink have been reported in the Netherlands and Denmark (Hammer et al., 2021; Oude Munnink et al., 2021). In the Netherlands, for the first 16 infected farms, 68% (66/97) of farm residents, workers, and their contacts became infected with a mink SARS-CoV-2 variant (Oude Munnink et al., 2021). In Denmark, sequencing of 10,386 human samples revealed SARS-CoV-2 mink-variants in 750 (7.2%; https://www.who.int/csr/don/03-december-2020-mink-associated-sars-cov2-denmark/en/, Accessed February 03, 2021). In Denmark, SARS-CoV-2 circulates rapidly in mink farms and human communities close to farms, and 40% of human cases of COVID-19 in the North Jutland Region are with mink variants (Larsen and Paludan, 2020). In this area, five different related clusters with several mutations in the spike protein have been identified. In particular, one variant, “Cluster 5,” has caused alarm, as four changes in the spike protein sequence were detected (Larsen and Paludan, 2020). Twelve human cases of infection with “Cluster 5” were identified in September 2020 in the North Jutland area. Among them, eight had a connection to a mink farm and four were from the local community (Larsen and Paludan, 2020).

Obviously, the risk of transmission of mink SARS-CoV-2 to humans is greatly increased when there are large numbers of infected animals in small spaces. An infected human can spread the mink SARS-CoV-2 variant in human populations. There is also a residual risk with the transport of live mink (1% of the annual production, mainly breeding animals, are transported live) which can contribute to viral spreading between farms, as well as the release of farmed mink into the wild by animal welfare activists. Infected mink released in the wild can infect other species, including domestic species such as cats and dogs. Fearful of seeing SARS-CoV-2 variants selected in mink such as “Cluster 5” spread more easily among people, and to be more deadly or to have a negative impact on the deployment of anti-COVID-19 vaccines, the Danish Government decided to cull 17 million farmed mink (Frutos and Devaux, 2020; Koopmans, 2021). Several countries (Spain, the Netherlands, and France) have also ordered the destruction of mink colonies infected with SARS-CoV-2.

## Discussion

Many species of animals can be infected with this emerging zoonosis. However, after 12 months of pandemic, among all types of farming, anthropo-zoonotic outbreaks have only been reported in mink farms. In mink farms, contagion is facilitated by the close proximity of animals and their low genetic diversity. They can therefore constitute a reservoir where the virus can mutate. Infected mink can possibly be asymptomatic carriers and transmit the SARS-CoV-2 (or new variants of this virus) to humans or animals living near farms. The discovery of a case of COVID-19 in a wild mink in the United States raises questions about the sustainability of a wild reservoir of SARS-CoV-2.

Currently, SARS-CoV-2 outbreaks or cases in farmed mink have been reported all over the world, except in China and Russia. While the biggest European producers, Dutch and Danish, have been ordered by their health authorities to kill their farmed mink and ban their breeding, Russia has targeted the development of a vaccine for mink to prevent the spread of COVID-19.^1^ Indeed, it seems that the Federal Service for Veterinary and Phytosanitary Supervision developed a vaccine for animals that is currently being tested (https://www.vedomosti.ru/society/news/2020/12/11/850628-rosselhoznadzor-nazval-datu-sozdaniya-vaktsini, Accessed February 03, 2021).

Overall, the rapid spread of SARS-CoV-2 in farmed mink raises many questions. First, there are questions concerning a potential role in the early stages of the pandemic, especially as an intermediate host. Then, there are questions concerning the rapid spread of a new virus to a new host, leading to an accumulation of mutations with a potential impact on: (1) the fitness of the virus; (2) its contagiousness; (3) its pathogenicity; (4) reinfections with the different mutants generated; (5) effectiveness of immunotherapy; and (6) the effectiveness of vaccines.

## Author Contributions

FF, OM, P-EF, and DR contributed to conception and design of the study. FF, OM, MM, CD, PC, AL, P-EF, and DR analyzed and interpreted the data. FF, OM, MM, CD, PC, AL, and P-EF wrote sections of the manuscript. All authors contributed to the article and approved the submitted version.

### Conflict of Interest

The authors declare that the research was conducted in the absence of any commercial or financial relationships that could be construed as a potential conflict of interest.

## Footnotes

^1^https://furcommission.com/
